# Single-Step Organization of Plasmonic Gold Metamaterials with Self-Assembled DNA Nanostructures

**DOI:** 10.34133/2019/7403580

**Published:** 2019-08-21

**Authors:** Shaokang Ren, Jun Wang, Chunyuan Song, Qian Li, Yanjun Yang, Nan Teng, Shao Su, Dan Zhu, Wei Huang, Jie Chao, Lianhui Wang, Chunhai Fan

**Affiliations:** ^1^Key Laboratory for Organic Electronics and Information Displays (KLOEID), Institute of Advanced Materials (IAM), Jiangsu Key Laboratory for Biosensors, School of Materials Science and Engineering, Nanjing University of Posts and Telecommunications, Nanjing 210023, China; ^2^School of Chemistry and Chemical Engineering and Institute of Molecular Medicine, Renji Hospital, School of Medicine, Shanghai Jiao Tong University, Shanghai 200240, China

## Abstract

Self-assembled DNA nanostructures hold great promise as nanoscale templates for organizing nanoparticles (NPs) with near-atomistic resolution. However, large-scale organization of NPs with high yield is highly desirable for nanoelectronics and nanophotonic applications. Here, we design five-strand DNA tiles that can readily self-assemble into well-organized micrometer-scale DNA nanostructures. By organizing gold nanoparticles (AuNPs) on these self-assembled DNA nanostructures, we realize the fabrication of one- and two-dimensional Au nanostructures in single steps. We further demonstrate the one-pot synthesis of Au metamaterials for highly amplified surface-enhanced Raman Scattering (SERS). This single-step and high-yield strategy thus holds great potential for fabricating plasmonic metamaterials.

## 1. Introduction

Multiple-dimensional gold (Au) nanostructures such as one-dimensional (1D) nanowires [1–4], two-dimensional (2D) nanolattice [5–11], and three-dimensional (3D) crystalline lattices [12, 13] have attracted intense attention because of their potential applications in nanoelectronics [14], nanophotonics [4, 10, 15, 16], and nanosensors [17, 18]. Compared with top-down approaches like electron-beam lithography [19, 20] and focused-ion beam etching, DNA directed bottom-up self-assembly holds great promise to build well-organized Au nanostructures with programmable design, cost-effectiveness, and scalable synthesis [21, 22].

Structural DNA nanotechnology offers unprecedented opportunities for assembling Au nanostructures [11]. During the past two decades, numerous DNA nanostructures with various shapes in multiple dimensions were fabricated with one-pot assembly [23–25]. Particularly, the DNA origami techniques [26], which fold long single-stranded DNA into desired shapes, allow for creation of custom-designed and discrete DNA nanostructures. Because of the nanometer-scale precision and full addressability, DNA nanostructures are employed as templates [16, 27, 28] or linkers to organize Au nanoparticles (NPs) into multidimensional discrete nanostructures, lines, lattices, and crystals [29–31]. The typical procedure to create these AuNPs nanostructures usually contains two steps: assembly of DNA templates and organization of AuNPs, which would take several hours and even days [2, 9, 10]. These studies have mostly focused on studies of versatile strategies to fabricate complex plasmonic architectures with multiple functions or tailored optical response. Despite the rapid progress, a robust design strategy of DNA nanostructures together with timesaving protocols for AuNPs organization remains highly desirable [32–36]. Here, we demonstrate the realization of plasmonic Au metamaterials through one-pot assembly of AuNPs with five DNA strands and then investigate their optical applications.

## 2. Results

**Figure 1:**
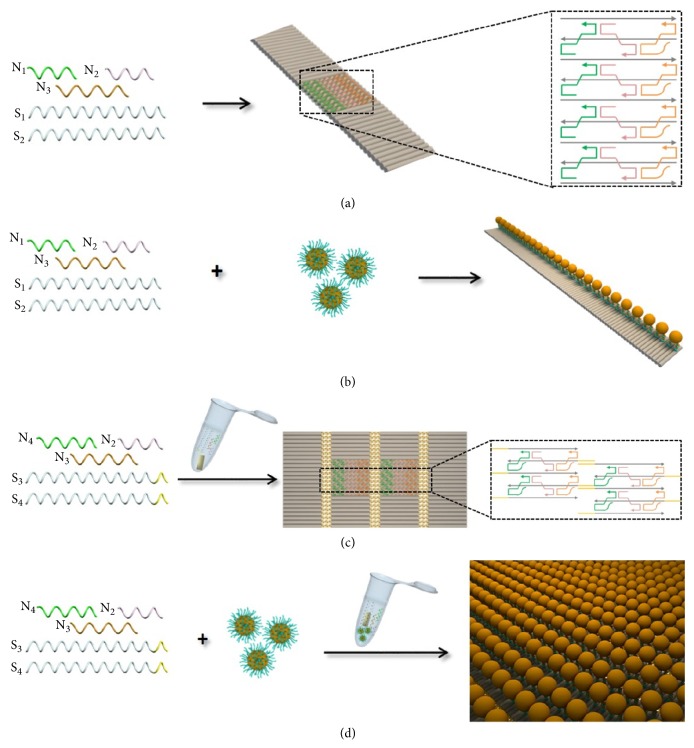
Schematic illustration of the one-pot self-assembly of DNA nanostructures and plasmonic metamaterials. (a) RDNs. (b) AuNPs lines. (c) 2D DNA lattices assisted by mica. (d) AuNPs lattices assisted by mica.

To assemble plasmonic gold metamaterials, ribbon-like DNA origami nanostructures (RDNs) were used as templates, in which three shorter DNA strands (N_1_, N_2_, and N_3_) not only fold two longer DNA strands (S_1_ and S_2_) into repeated rectangular units but connect neighbor units into long ribbons (Figure 1(a)). By extending N_3_ with a capture sequence as a binding site, AuNPs functionalized with thiol-DNA (SH-DNA-AuNPs) are mixed together with five strands in one pot to generate one-dimensional (1D) AuNPs lines. This strategy is also applied to organize gold nanorods (AuNRs) which functionalized with thiol-DNA (SH-DNA-AuNRs) into 1D AuNRs lines (Figure 1(b)). To obtain two-dimensional (2D) DNA lattices, S_1_ and S_2_ are extended with complementary overhangs of 15 bases into S_3_ and S_4_. Assisted by mica surface, five strands (N_2_, N_3_, N_4_, S_3_, and S_4_) are directly assembled into 2D lattices (Figure 1(c)). Using these lattices which have two binding sites on N_3_ and N_4_ as templates, 2D AuNPs lattices can be directly assembled with five strands in one pot assisted by mica (Figure 1(d)).

**Figure 2:**
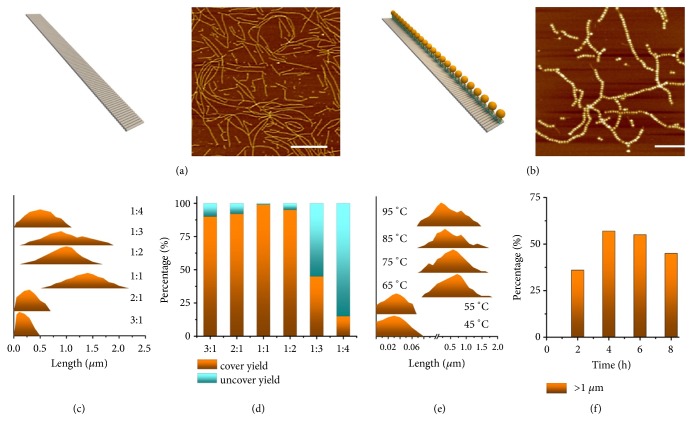
(a, b) Schematic illustrations and AFM images of 1D RDNs and 5 nm-sized AuNPs lines, scale bars: 500 nm. (c, d) Length density plots and coverage rate histogram of 5 nm-sized AuNPs lines on different ratios of AuNPs to stand N_3_. (e) Density plots of 5 nm-sized AuNPs lines on different initial temperature in annealing process. (f) Histogram of the percentage of micrometer-long AuNPs lines in different annealing time.

With the optimization of sequences for N_1_, N_2_, N_3_, S_1_, and S_2_ by SEQUIN [37], we surprisingly found that five DNA strands can self-assemble into RDNs in 20 minutes at room temperature in 1×TAE/Mg^2+^ buffer. AFM measurements showed that the lengths of RDNs were ranging from hundreds of nanometers to several micrometers and the width of RDNs was ~17 nm, which is in agreement with its theoretical value (Figures 2(a) and [Supplementary-material supplementary-material-1]). Using one-pot assembly strategy, we generated AuNPs lines with a high coverage rate up to 95% by simply mixing 5 nm-sized SH-DNA-AuNPs with five DNA strands (Figures 2(b) and [Supplementary-material supplementary-material-1]). To better understand the assembly process, we employed the two-step protocol to generate the AuNPs lines; that is, the RDNs templates were firstly assembled followed by anchoring of SH-DNA-AuNPs onto template surfaces. The coverage rate of thus assembled AuNPs lines was 45% ([Supplementary-material supplementary-material-1]), which is significantly lower than the one obtained with the one-pot assembly strategy. In the one-pot assembly process, SH-DNA-AuNPs might hybridize with strand N_3_ that followed by folding with other strands into lines. While in the two-step assembly, the negative charged DNA nanoribbon may hinder the hybridization of SH-DNA-AuNPs to N_3_. In theory, the gap between neighboring N_3_ on the template is ~ 6 nm, which is big enough to anchor 5 nm-sized AuNPs.

Different experimental conditions were systematically investigated. Micrometer-long AuNPs lines could be assembled with 99% coverage rate (Figures 2(c), 2(d), and [Supplementary-material supplementary-material-1]), with the optimized 1:1 concentration ratio of SH-DNA-AuNPs to N_3_. Considering that the RDNs can be easily assembled at room temperature, different initial temperatures for one-pot assembly were studied. When the initial temperature was equal to or higher than 65°C, micrometer-long AuNPs lines could be formed (Figures 2(e) and [Supplementary-material supplementary-material-1]). We also monitored the annealing time for one-pot assembly when the initial temperature was 65°C. After 2 h annealing, the percentage of micrometer-long AuNPs lines in the product was 36%, which was increased to 57% as prolonging the annealing time to 4 h (Figures 2(f) and [Supplementary-material supplementary-material-1]). We also examined the thermal stability of the 5 nm-sized AuNPs lines by keeping the samples in water bath with different temperature for 20 min. The AFM imaging results indicated that the structures were stable when they were heated to 40°C ([Supplementary-material supplementary-material-1]).

**Figure 3:**
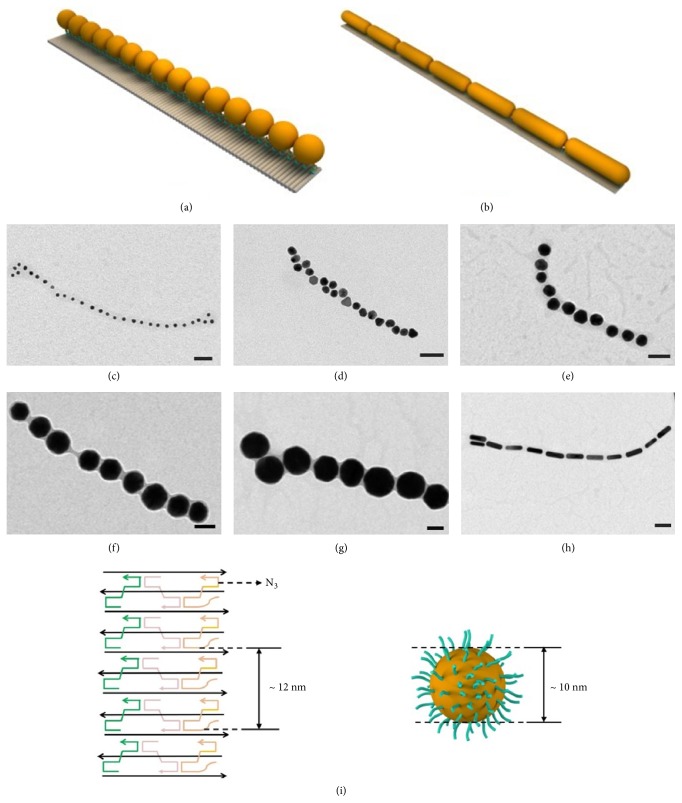
Schematic illustrations (a) and TEM images of 10 nm- (c), 20 nm- (d), 30 nm- (e), 50 nm- (f), and 80 nm-sized (g) AuNPs lines. Schematic illustrations (b) and TEM images of 20×70 nm-sized AuNRs lines (h). Scale bars: 50 nm. Four strands of N_3_ with other DNA strands form an 18 nm-long unit of nanoribbon which are adapted to the size of 20 nm-sized AuNPs (i).

We further applied the one-pot assembly strategy to organize AuNPs of different sizes (Figure 3). TEM images validated the one-pot assembly of 10 nm-, 20 nm-, 30 nm-, 50 nm-, and 80 nm-sized AuNPs lines (Figures 3(c)–3(g) and [Supplementary-material supplementary-material-1]), which were obtained by annealing five strands with SH-DNA-AuNPs in 1×TAE/Mg^2+^ buffer from 65°C to 25°C in 4 h. To obtain high-quality 1D AuNPs lines, the concentration ratio of SH-DNA-AuNPs to N_3_ was optimized for AuNPs of different sizes. For example, 4 strands of N_3_ with other DNA strands form an 18 nm-long unit of nanoribbon, which are adapted to the size of 20 nm-sized AuNPs (Figure 3(i)), and the concentration ratio of 20 nm-sized AuNPs to N_3_ was 1:4. Correspondingly, the concentration ratio of SH-DNA-AuNPs to N_3_ was 1:2 and 1:6 for 10 nm- and 30 nm-sized AuNPs, 1:10 and 1:16 for 50 nm- and 80 nm-sized AuNPs, respectively. 

With the increase of the size of AuNPs, the length of the AuNPs lines decreased from micrometers to hundreds of nanometers. These phenomena were adapted to the supposition we mentioned above; that is, SH-DNA-AuNPs may firstly hybridize with N_3_; therefore, it is more difficult for SH-DNA-AuNPs-N_3_ of larger sizes to further hybridize with other strands to form lines. Using the same one-pot assembly strategy, AuNRs (20×70 nm) were organized to form AuNRs lines with the concentration ratio of SH-DNA-AuNRs to N_3_ of 1:14, confirmed by TEM imaging results (Figures 3(h) and [Supplementary-material supplementary-material-1]).

**Figure 4:**
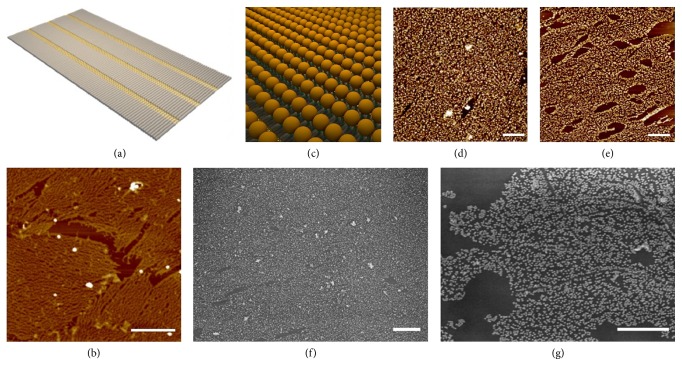
Schematic illustrations (a) and AFM image (b) of 2D DNA lattices. Schematic illustrations (c) and AFM image of 5 nm- (d) and 10 nm-sized (e) AuNPs lattices, scale bars: 500 nm. SEM images of 20 nm-sized (f) and 30 nm-sized (g) AuNPs lattices, scale bars: 1 *μ*m.

To widen the range of one-pot assembly strategy, we extended S_1_ and S_2_ with complementary 15-base overhangs. Five strands (N_2_, N_3_, N_4_, S_3_, and S_4_) were mixed together in 1×TAE/Mg^2+^ buffer and annealed from 65°C to 25°C for 12 h. Although there were some connections between RDNs, network-like DNA nanostructures other than 2D lattices were formed, demonstrated by AFM imaging results ([Supplementary-material supplementary-material-1]). When mica was annealed together with five strands, the Mg^2+^ in buffer helped DNA strands to absorb onto mica surface and 2D DNA lattices were generated, which is validated by AFM images (Figures 4(a) and 4(b)). The growth processes of 2D lattices were proposed to be assisted by mica; that is, earlier formed RDNs could adsorb on negatively charged mica surface as nuclei and then single strands or smaller RDNs diffuse onto the surface for 2D crystallization. Using the one-pot assembly strategy, SH-DNA-AuNPs could be assembled into 2D AuNPs lattices assisted by mica (Figure 4(c)). AFM and SEM images demonstrated the formation of different-sized AuNPs 2D lattices (Figures 4(d)–4(g), [Supplementary-material supplementary-material-1], and [Supplementary-material supplementary-material-1]).

**Figure 5:**
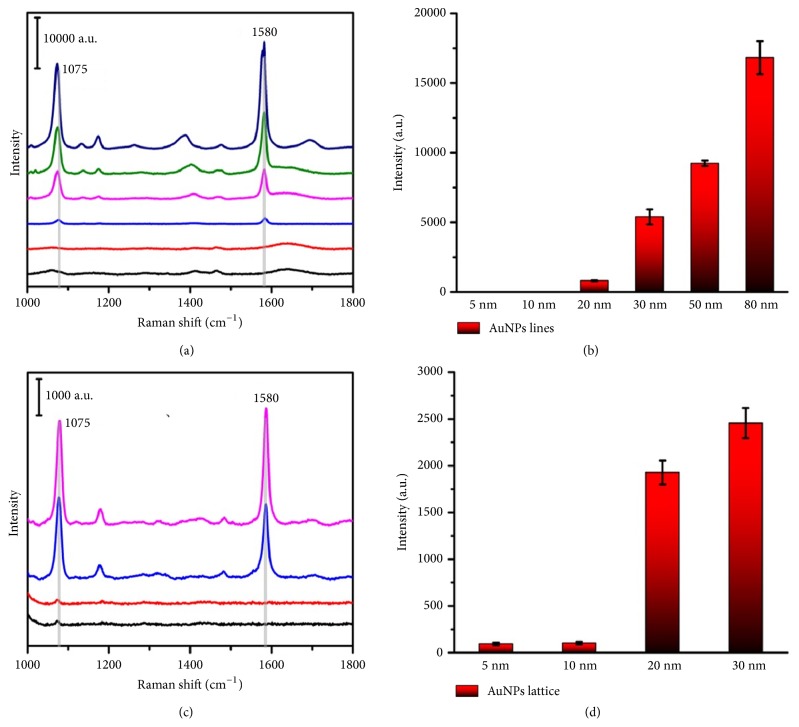
Typical SERS spectra of 1D AuNPs lines and 2D AuNPs lattices. (a) 5 nm- (black), 10 nm- (red), 20 nm- (blue), 30 nm- (purple), 50 nm- (green), and 80 nm-sized AuNPs lines (dark blue) with 4-MBA as the SERS reporter molecule. (b) Changes of the characteristic SERS intensity at 1075 cm^−1^ of different-sized AuNPs lines. (c) 5 nm- (black), 10 nm- (red), 20 nm- (blue), and 30 nm-AuNPs lattices (purple) with 4-MBA as the SERS reporter molecule. (d) Changes of the characteristic SERS intensity at 1075 cm^−1^ of different-sized AuNPs lattices.

**Figure 6:**
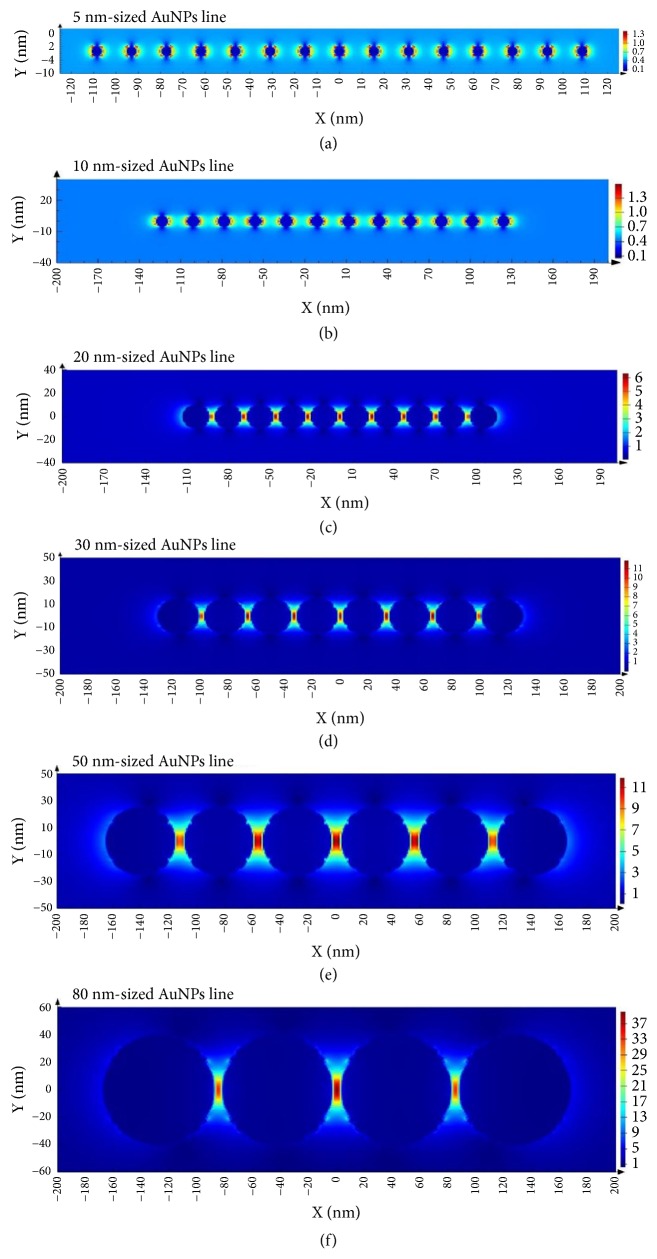
Finite-difference time-domain (FDTD) simulation of the electromagnetic field distributions of the different-sized AuNPs line. The gap between AuNPs is 10.6 nm (a), 12.7 nm (b), 2.7 nm (c), 2.8 nm (d), 5.9 nm (e), and 5.5 nm (f), respectively. The incident light is along x axis direction.

Because metal nanostructures can excite collectively coupled plasmons when in interacting with light, [15, 16] AuNPs metamaterials impelled us to consider their potential plasmonic properties [27, 38]. Previous studies showed that proper distances between individual AuNP could generate hot spots that induce stronger SERS signals. As most of the distances between the AuNPs in our 1D AuNPs lines and 2D AuNPs lattices were less than 10 nm, their coupling of the plasmons was detected by UV-vis absorption ([Supplementary-material supplementary-material-1]). We used 4-MBA as a Raman-active molecule which could covalently attach to AuNPs via Au-S bond. The frequencies of 1580 cm^−1^ and 1075 cm^−1^ in the SERS spectra were attributed to 4-MBA. SERS signals were hardly observed from 5 nm- and 10 nm-sized AuNPs lines or lattices, because of the weak coupling of the larger gaps between small-sized AuNPs. However, large-sized AuNPs lines and lattices both induced enhanced SERS signals. Take the peak at 1075 cm^−1^ for an example; an enhanced signal generated by 80 nm-sized AuNPs lattices was twenty times higher than that of 20 nm-sized AuNPs lines (16833.93 to 804.98 a.u. at 1075 cm^−1^, Figure 5). Based on Maxwell's curl equations, the electrical field in different-sized AuNPs line upon illumination (Figure 6) was simulated by the comprehensive finite difference time domain (FDTD) method. On this basis, the enhancement could be naturally interpreted by the increasing of the hotspots created in the different-sized AuNPs lines. The same enhanced phenomenon was observed when comparing 20 nm- and 10 nm-sized AuNPs lattices.

## 3. Discussion

We designed five-strand DNA tiles for the fabrication of DNA nanostructures in micrometer scale, which facilitate the realization of 1D and 2D plasmonic gold metamaterials. Using one-pot assembly strategy, AuNPs of different sizes are organized for high-quality 1D lines and 2D lattices, which could generate enhanced Raman scattering. This strategy is of great importance for single-step, rapid, and high-yield assembly of AuNPs into plasmonic metamaterials. As semiconductor and information technologies approach their physical limits, it is a great challenge to combine the functionality of biological systems and semiconductors. Our strategy would provide a potential tool to deal with interfacing biological materials with conventional semiconductors for practical applications.

## 4. Materials and Methods

### 4.1. Preparation of SH-DNA-AuNPs/AuNRs Conjugates

5 *μ*L of 100 *μ*M SH-DNA and 100 *μ*L of 83.3 nM 5 nm-sized spherical AuNPs were mixed together in 0.5 × TBE buffer (89 mM Tris, 89 mM boric acid, 2 mM EDTA, pH 8.0), which was incubated at 37°C for about 4 h. 10 *μ*L 3 M NaCl was gradually added to reach a final concentration of 0.3 M NaCl for aging. The solution was kept in 37°C overnight. To remove excess SH-DNA, the solution was centrifuged (12000 rpm, 25 min) and the supernatant was carefully removed by pipette. The SH-DNA-AuNPs conjugates were washed by 0.5 × TBE buffer for three times. For spherical AuNPs of different diameters, the protocols were identical except that the ratios of 10 nm-, 20 nm-, 30 nm-, 50 nm-, and 80 nm-sized AuNPs to DNA were approximately 1:300. For 20 × 70 nm gold nanorods (AuNRs), excess amounts of cetyltrime-thyl ammonium bromide in the AuNRs solution had to be removed by centrifugation at 4000 rpm for 10 min and the ratio of AuNPs to DNA was approximately 1:600.

### 4.2. One-Pot Assembly of RDNs, AuNPs, and AuNRs Lines

Stock solutions (5 *μ*M) of DNA single strand (N_1_, N_2_, N_3_, S_1_, and S_2_) were mixed together (2 *μ*L for each strand) in 20 minutes at room temperature. The RDNs were obtained with a final concentration of 500 nM in TAE buffer (40 × 10^−3^ M Tris, 20 × 10^−3^ M acetic acid, 2 × 10^−3^ M EDTA, and 12.5 × 10^−3^ M magnesium acetate, pH 8.0). For 5 nm-sized AuNPs lines, 5 *μ*M ssDNA stock solutions (N_1_, N_2_, N_3_, S_1_, and S_2_, 2 *μ*L for each) and SH-DNA-AuNPs (the ratio of SH-DNA-AuNPs to strand N_3_ was 1:1) were mixed together and annealed from 65°C to 25°C for 4 h. The protocols for AuNPs lines of different diameters were identical except that the ratios of 10 nm-, 20 nm-, 30 nm-, 50 nm-, and 80 nm-sized AuNPs to N_3_ were 1:2, 1:4, 1:6, 1:10, and 1:16. For the AuNRs lines, the ratio of 20 × 70 nm AuNRs to N_3_ was 1:14.

### 4.3. One-Pot Assembly of Two-Dimensional (2D) DNA and AuNPs Lattices

5 *μ*M ssDNA stock solutions (N_2_, N_3_, N_4_, S_3_, and S_4_, 2 *μ*L for each) were mixed together with mica in TAE buffer. The mixture was annealed in 2L water from 65°C to 25°C for 12 h to assemble 2D DNA lattices. For 5 nm-sized AuNPs lattices, 5 *μ*M ssDNA stock solutions (N_2_, N_3_, N_4_, S_3_, and S_4_, 2 *μ*L for each) and SH-DNA-AuNPs (the ratio of SH-DNA-AuNPs to N_3_ was 1:1) were mixed together with mica in TAE buffer and annealed from 65°C to 25°C for 12 h. The protocols for AuNPs lattices of different sizes were identical.

### 4.4. AFM Characterization

5 *μ*L of RDNs or AuNPs lines was deposited onto the fresh cleaved mica, allowing for 3-5min adsorption. The samples were rinsed with pure water, dried under nitrogen, and then scanned in tapping mode by MultiMode 8 AFM with NanoScope V Controller (Bruker, Inc.).

### 4.5. TEM Characterization

For TEM imaging, 10 *μ*L of the sample was dropped on a carbon-coated grid (400 mesh; Ted Pella, Inc.). After 15 min deposition, the excess amounts of solution drop on the grid were wicked by a drop of water and then adsorbed into a filter paper. TEM images were obtained by a Tecnai G2-20S TWIN system, which was operated at 100 kV in a bright-field mode.

### 4.6. SEM Characterization

2D AuNPs lattices on the mica were washed by pure water and dried under nitrogen. The samples were rinsed with positively charged nanogold solution for 15 s to increase the conductivity. Then the mica was stuck to the sample table by conductive tape. SEM imaging was performed using a HITACHI S4800 at 80 kV in dark field mode.

### 4.7. SERS Characterization

Samples of AuNPs lines were incubated in 1 mM 4-MBA ethanolic solution for 2-3 h. 25 mL of the sample was deposited on a silicon substrate and performed in solution for SERS characterization. 10 *μ*L 10 mM 4-MBA ethanolic solution was deposited onto the mica surfaces with 2D AuNPs lattices for over 3 h, followed by a thorough rinse with pure ethanol. The SERS characterization was performed on mica surface. For all measurements, the confocal Raman microscope (Invia, Renishaw, England) was employed with a 633 nm He-Ne laser at the power of 0.08 mW. Other experimental parameters were as follows: objective 20×, acquisition time 10 s, slit of 100 *μ*m, resolution grating of 1800 grooves, and spectra ranged from 1000 to 1800 cm ^−1^.

## Data Availability

All data is available in the main text or the Supplementary Materials.

## Supplementary Materials

Supplementary Materials Supplementary Figure 1: schematic illustrations and AFM images of 1D RDNs. (a) The design of RDNs is that two 48nt sequences are folded by three staple strands into a ribbon like DNA nanostructure. (b) AFM image of RDNs. Scale bar: 500 nm. (c) AFM height and width images with corresponding line scans (inset of (b)) for bare RDNs. (d) Histogram of RDNs length counts. Supplementary Figure 2: 1D AuNPs lines. Schematics (a, b) and AFM images (c, d) are single- and two-step assembly of 5 nm-sized AuNPs lines, respectively. Scale bars: 500 nm. (e) Coverage rate histogram of single- and two-step assembly of AuNPs lines. Supplementary Figure 3: analysis and AFM images of 5 nm-sized AuNPs lines at different annealing temperatures. (a) 45°C. (b) 55°C. (c) 65°C. (d) 75°C. (e) 85°C. (f) 95°C. Scale bars: 200 nm. Supplementary Figure 4: analysis and AFM images of 5 nm-sized AuNPs lines at different ratio of AuNPs to RDNs when annealing temperature is 65°C. (a) 3:1. (b) 2:1. (c) 1:1. (d) 1:2. (e) 1:3. (f) 1:4. Scale bars: 200 nm. Supplementary Figure 5: annealing temperature is 65°C and the ratio of AuNPs to RDNs is 1:1; analysis and AFM images of 5 nm-sized AuNPs lines with annealing time. (a) 0.1 h. (b) 0.5 h. (c) 1 h. (d) 1.5 h. (e) 2 h. (f) 4 h. (g) 6 h. (h) 8 h. Scale bars: 200 nm. Supplementary Figure 6: AFM images of 5 nm-sized AuNP lines which were heated to 30°C, 40°C, 50°C, and 60°C. Scale bars: 200 nm. Supplementary Figure 7: schematic illustrations and more TEM images of organized different sizes of AuNPs lines. (a) 10 nm-sized AuNPs lines. (b) 20 nm-sized AuNPs lines. (c) 30 nm-sized AuNPs lines. (d) 50 nm-sized AuNPs lines. (e) 80 nm-sized AuNPs lines. (f) 20×70 nm-sized AuNRs lines. Scale bars: 50 nm. Supplementary Figure 8: AFM images of 2D DNA lattices without mica surface-mediated and height and width image with corresponding line scans (inset of (c)) for 2D DNA lattices. Supplementary Figure 9: AFM images of 2D DNA lattices (a) and 2D AuNPs lattices of mica surface-mediated. (b) 5 nm-sized AuNPs lattices. (c) 10 nm-sized AuNPs lattices. SEM images of 20 nm-sized (d) and 30 nm-sized (e) AuNPs lattices. Supplementary Figure 10: AFM images of 5 nm-sized SH-DNA-AuNPs (a) and 2D DNA lattices (b) and 5 nm-sized AuNPs lattices. Scale bar: 50 nm. Supplementary Figure 11: UV–Vis characterization of different-sized AuNPs lines.
