# Supplementary material for: Single-Step Organization of Plasmonic Gold Metamaterials with Self-Assembled DNA Nanostructures
Source: Research (Wash D C). 2019 Aug 21;2019:7403580. doi: 10.34133/2019/7403580 (PMC6750079; doi:10.34133/2019/7403580)
Supplement: Supplementary Materials — Supplementary Figure 1: schematic illustrations and AFM images of 1D RDNs. (a) The design of RDNs is that two 48nt sequences are folded by three staple strands into a ribbon like DNA nanostructure. (b) AFM image of RDNs. Scale bar: 500 nm. (c) AFM height and width images with corresponding line scans (inset of (b)) for bare RDNs. (d) Histogram of RDNs length counts. Supplementary Figure 2: 1D AuNPs lines. Schematics (a, b) and AFM images (c, d) are single- and two-step assembly of 5 nm-sized AuNPs lines, respectively. Scale bars: 500 nm. (e) Coverage rate histogram of single- and two-step assembly of AuNPs lines. Supplementary Figure 3: analysis and AFM images of 5 nm-sized AuNPs lines at different annealing temperatures. (a) 45°C. (b) 55°C. (c) 65°C. (d) 75°C. (e) 85°C. (f) 95°C. Scale bars: 200 nm. Supplementary Figure 4: analysis and AFM images of 5 nm-sized AuNPs lines at different ratio of AuNPs to RDNs when annealing temperature is 65°C. (a) 3:1. (b) 2:1. (c) 1:1. (d) 1:2. (e) 1:3. (f) 1:4. Scale bars: 200 nm. Supplementary Figure 5: annealing temperature is 65°C and the ratio of AuNPs to RDNs is 1:1; analysis and AFM images of 5 nm-sized AuNPs lines with annealing time. (a) 0.1 h. (b) 0.5 h. (c) 1 h. (d) 1.5 h. (e) 2 h. (f) 4 h. (g) 6 h. (h) 8 h. Scale bars: 200 nm. Supplementary Figure 6: AFM images of 5 nm-sized AuNP lines which were heated to 30°C, 40°C, 50°C, and 60°C. Scale bars: 200 nm. Supplementary Figure 7: schematic illustrations and more TEM images of organized different sizes of AuNPs lines. (a) 10 nm-sized AuNPs lines. (b) 20 nm-sized AuNPs lines. (c) 30 nm-sized AuNPs lines. (d) 50 nm-sized AuNPs lines. (e) 80 nm-sized AuNPs lines. (f) 20×70 nm-sized AuNRs lines. Scale bars: 50 nm. Supplementary Figure 8: AFM images of 2D DNA lattices without mica surface-mediated and height and width image with corresponding line scans (inset of (c)) for 2D DNA lattices. Supplementary Figure 9: AFM images of 2D DNA lattices (a) and 2D AuNPs lattices of mica sur [file 7403580.f1.docx]

# Supporting

**Single-Step Organization of Plasmonic Gold Metamaterials with Self-Assembled DNA Nanostructures**

Shaokang Ren,^1,†^ Jun Wang,^1,†^ Chunyuan Song,^1^ Qian Li,^2^ Yanjun Yang, ^1^ Nan Teng, ^1^ Shao Su,^1^ Dan Zhu,^1^ Wei Huang, ^1^ Jie Chao,^1,^* Lianhui Wang,^1,^* and Chunhai Fan^2,^*

*^1^Key Laboratory for Organic Electronics and Information Displays (KLOEID), Institute of Advanced Materials (IAM), Jiangsu Key Laboratory for Biosensors, School of Materials Science and Engineering, Nanjing University of Posts and Telecommunications, Nanjing, 210023, China.*

*^2^School of Chemistry and Chemical Engineering, and Institute of Molecular Medicine, Renji Hospital, School of Medicine, Shanghai Jiao Tong University, Shanghai 200240, ChinaUse superscript numbers (1, 2, 3) to designate author affiliations.*

*Correspondence should be addressed to Jie Chao; iamjchao@njupt.edu.cn, Lianhui Wang; iamlhwang@njupt.edu.cn, and Chunhai Fan; fanchunhai@sjtu.edu.cn

†These authors contributed equally to this work


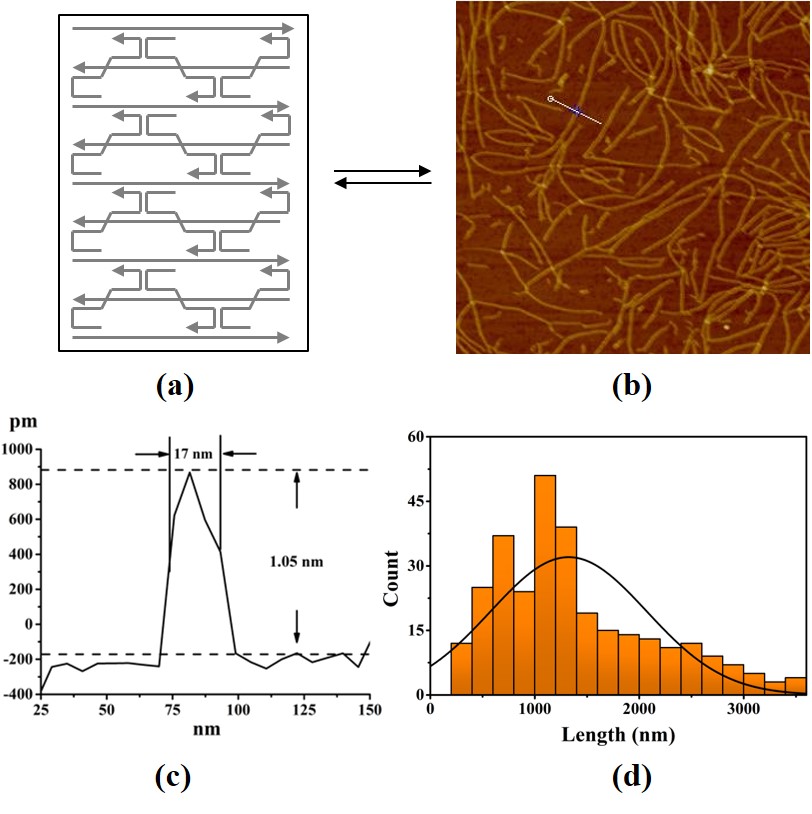


**FIGURE S1.** Schematic illustrations and AFM images of 1D RDNs. (a) The design of RDNs is that two 48nt sequences is folded by three staple strands into a ribbon like DNA nanostructure. (b) AFM image of RDNs. Scale bar: 500 nm. (c) AFM height and width images with corresponding line scans (inset of (b)) for bare RDNs. (d) Histogram of RDNs length counts.


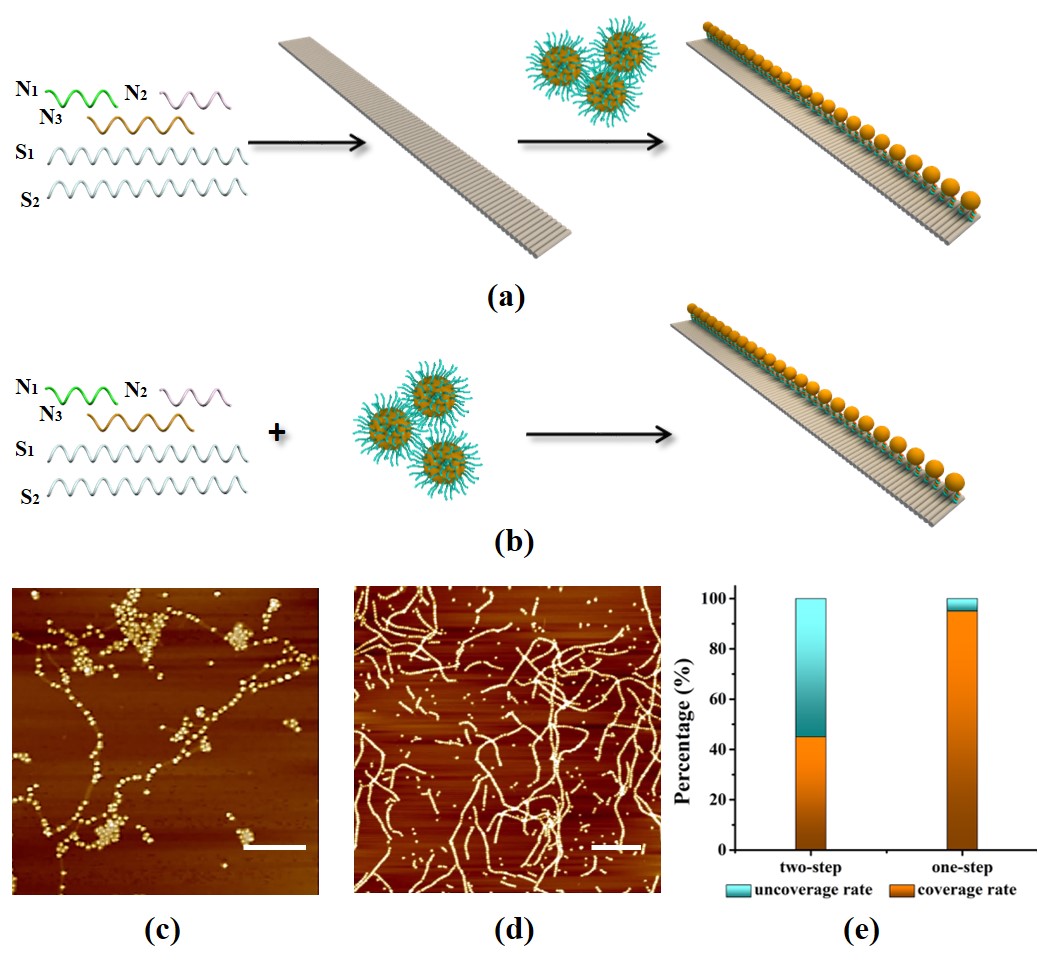


**FIGURE S2.** 1D AuNPs lines. Schematics (a, b) and AFM images (c, d) are single and two step assembly of 5 nm-sized AuNPs lines, respectively. Scale bars: 500 nm. (e) Coverage rate histogram of single and two step assembly of AuNPs lines.

**
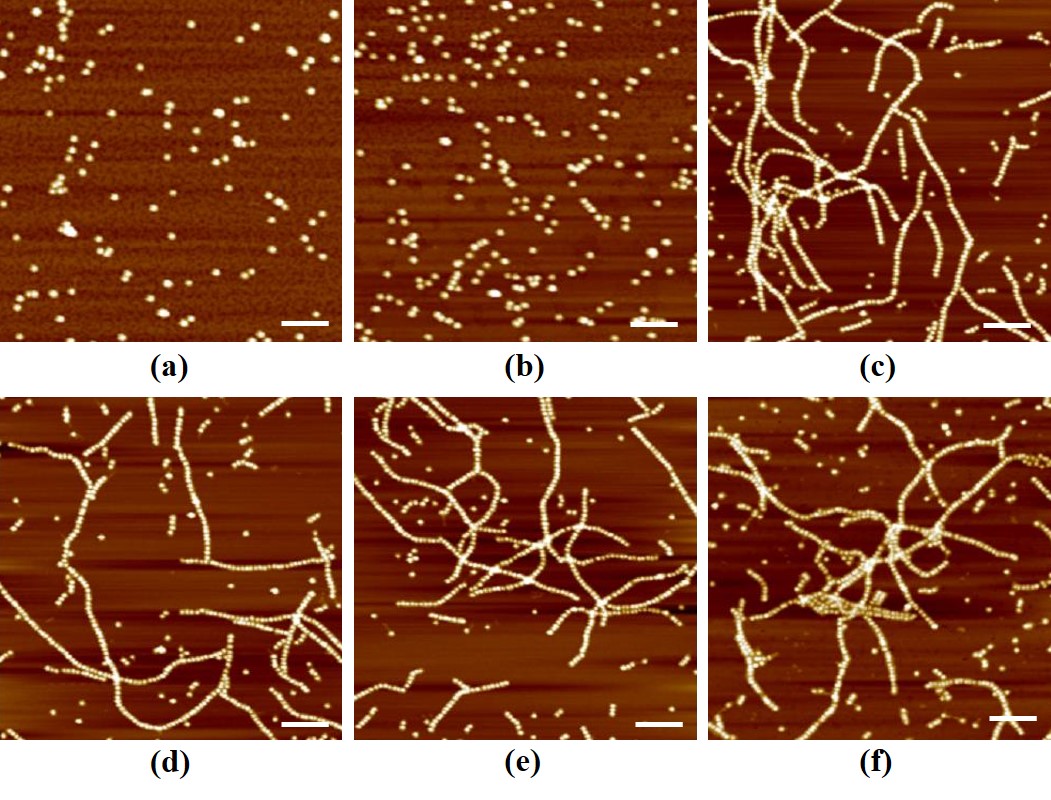
**

**FIGURE S3.** Analysis and AFM images of 5 nm-sized AuNPs lines at different annealing temperature. (a) 45 ̊C. (b) 55 ̊C. (c) 65 ̊C. (d) 75 ̊C. (e) 85 ̊C .(f) 95 ̊C. Scale bars: 200 nm.


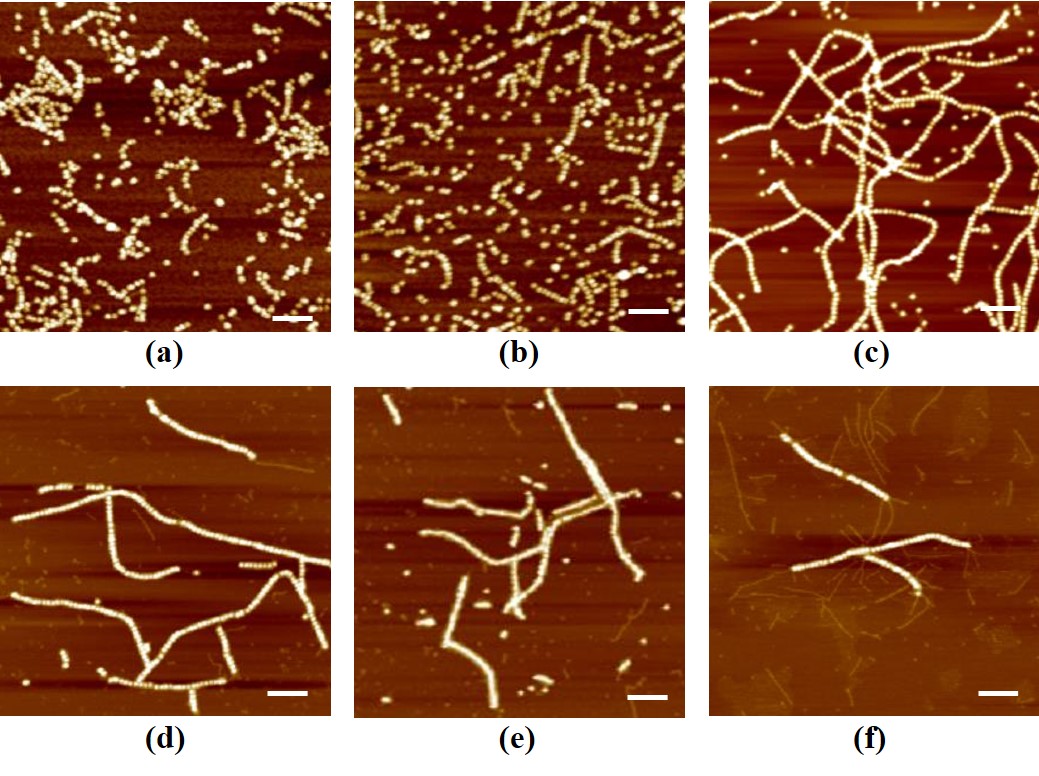


**FIGURE S4.** Analysis and AFM images of 5 nm-sized AuNPs lines at different ratio of AuNPs to RDNs when annealing temperature is 65 ̊C. (a) 3:1. (b) 2:1. (c) 1:1. (d) 1:2. (e) 1:3. (f) 1:4.Scale bars: 200 nm.


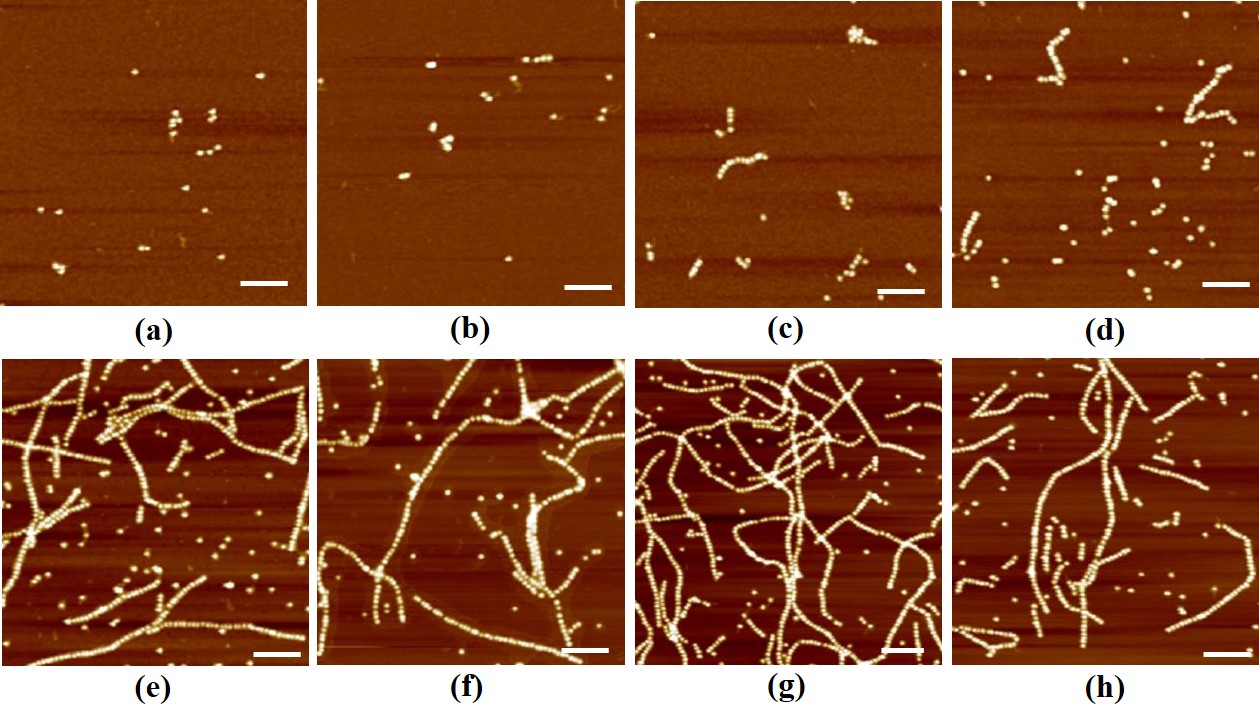


**FIGURE S5.** Annealing temperature is 65 ̊C and the ratio of AuNPs to RDNs is 1:1, analysis and AFM images of 5 nm-sized AuNPs lines with decreasing annealing time. (a) 0.1 h. (b) 0.5 h. (c) 1 h. (d) 1.5 h. (e) 2 h. (f) 4 h. (g) 6 h. (h) 8 h. Scale bars: 200 nm.


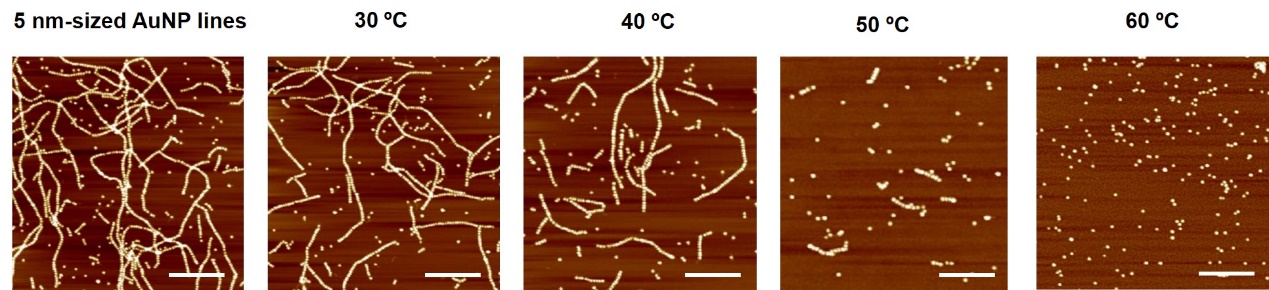


**FIGURE S6.** AFM images of 5 nm-sized AuNP lines which were heated to 30 ̊C, 40 ̊C, 50 ̊C and 60 ̊C. Scale bars: 200 nm.


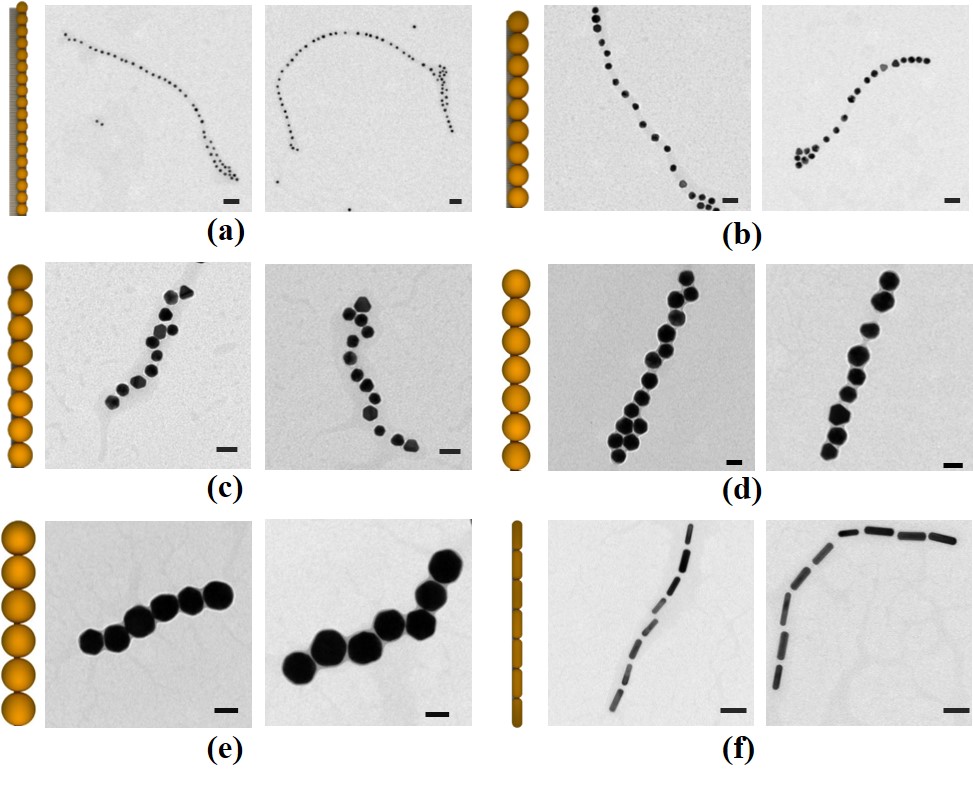


**FIGURE S7**. Schematic illustrations and more TEM images of organized different sized of AuNPs lines. (a) 10 nm-sized AuNPs lines. (b) 20 nm-sized AuNPs lines. (c) 30 nm-sized AuNPs lines. (d) 50 nm-sized AuNPs lines. (e) 80 nm-sized AuNPs lines. (f) 20×70 nm-sized AuNRs lines. Scale bars: 50 nm.


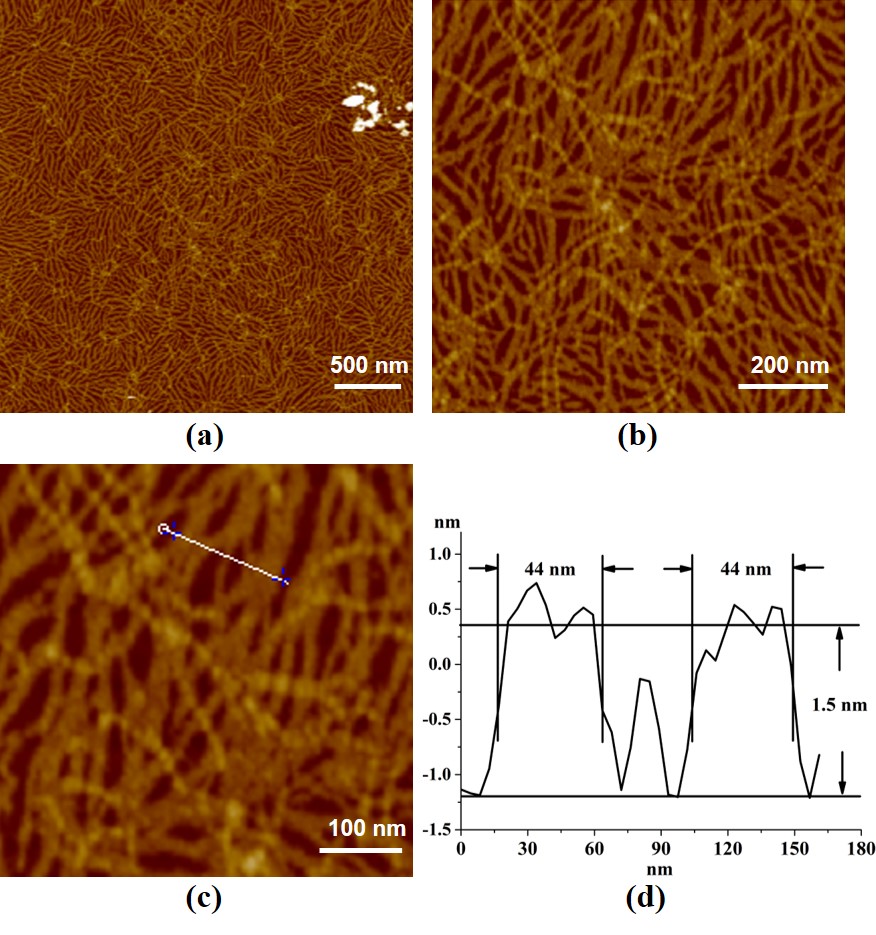


**FIGURE S8.** AFM images of 2D DNA lattices without mica surface-mediated and height and width image with corresponding line scans (inset of (c)) for 2D DNA lattices.


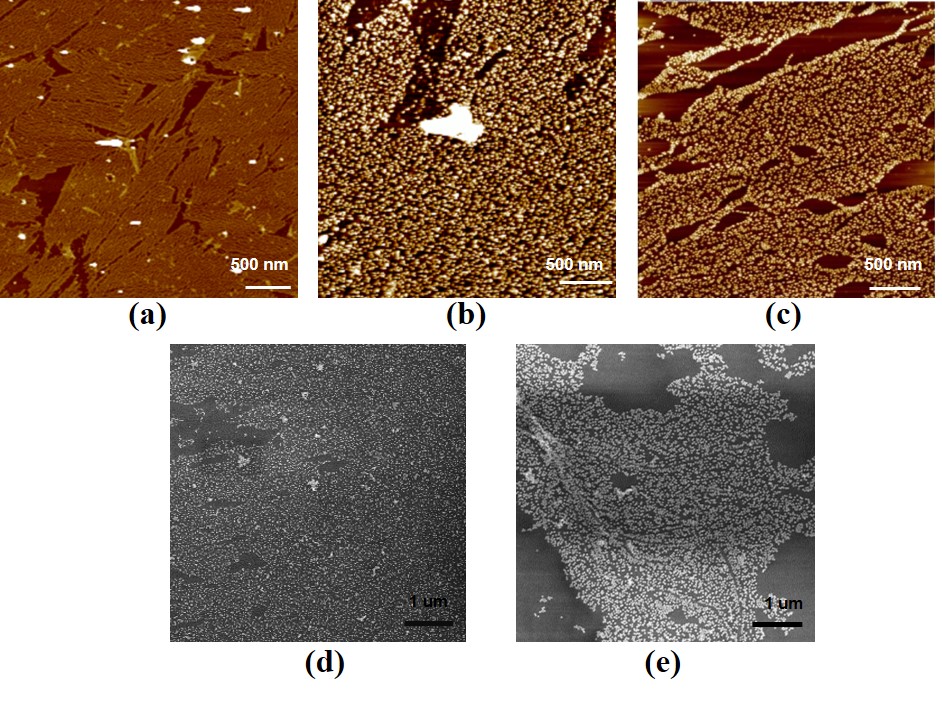


**FIGURE S9.** AFM images of 2D DNA lattices (a) and 2D AuNPs lattices of mica surface-mediated. (b) 5 nm-sized AuNPs lattices. (c) 10 nm-sized AuNPs lattices. SEM images of 20 nm-sized (d) and 30 nm-sized (e) AuNPs lattices.

The 2D AuNP lattices in the present study is not like random distribution. Before assembly, SH-DNA-AuNPs distributed randomly on mica surface (Figure S9(a)), the gap between which were ~20 nm based on AFM images. After one-pot assembly, AuNPs lattices were formed with the gap decreasing to ~6 nm (Figure S9(c)). These results clearly suggested that 2D AuNP lattices were formed with five DNA strands using the one-pot strategy.


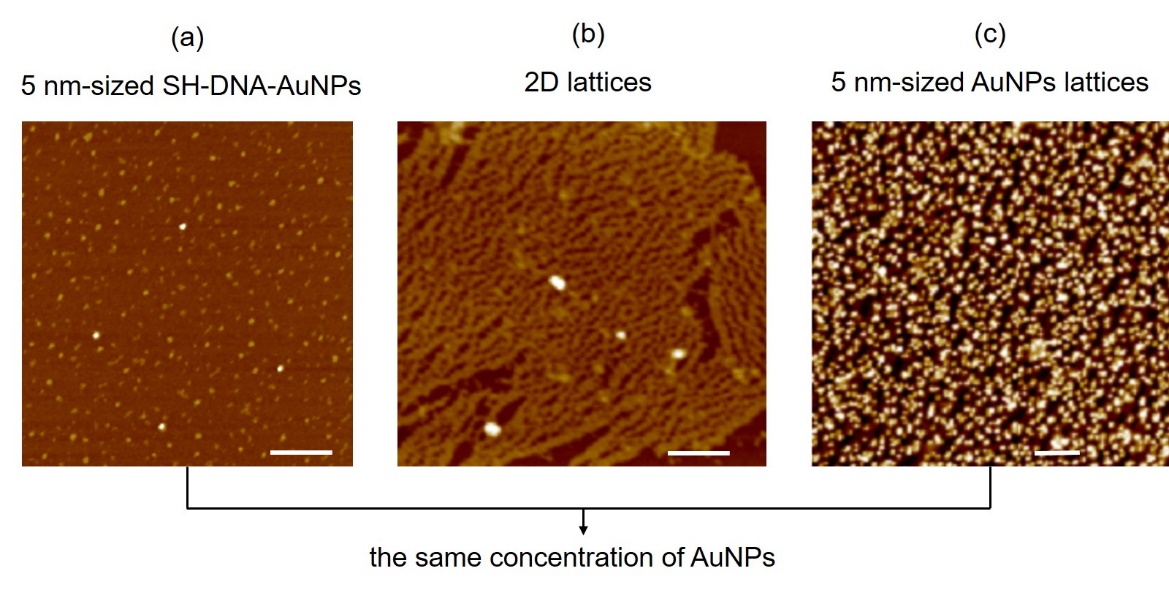


**FIGURE S10.** AFM images of 5 nm-sized SH-DNA-AuNPs (a), 2D DNA lattices (b) and 5 nm-sized AuNPs lattices. Scale bar: 50 nm.

UV–Vis Characterization of different sized AuNPs lines: Droplets (1 µL) samples of different sized pure AuNPs and AuNPs lines were measured in a 1 mm lightpath UV cell at room temperature using a NanoPhotometer RP-Class P330 three times and its wavelength range is 300 to 900 nm, respectively. The means of the read out values were calculated, which were used to draw images in Origin8. We carried out UV-vis spectral analysis of different sized AuNPs lines described above, and observed red shifts of the plasmonic resonance peak, which were ranging from 6 nm to 9 nm.


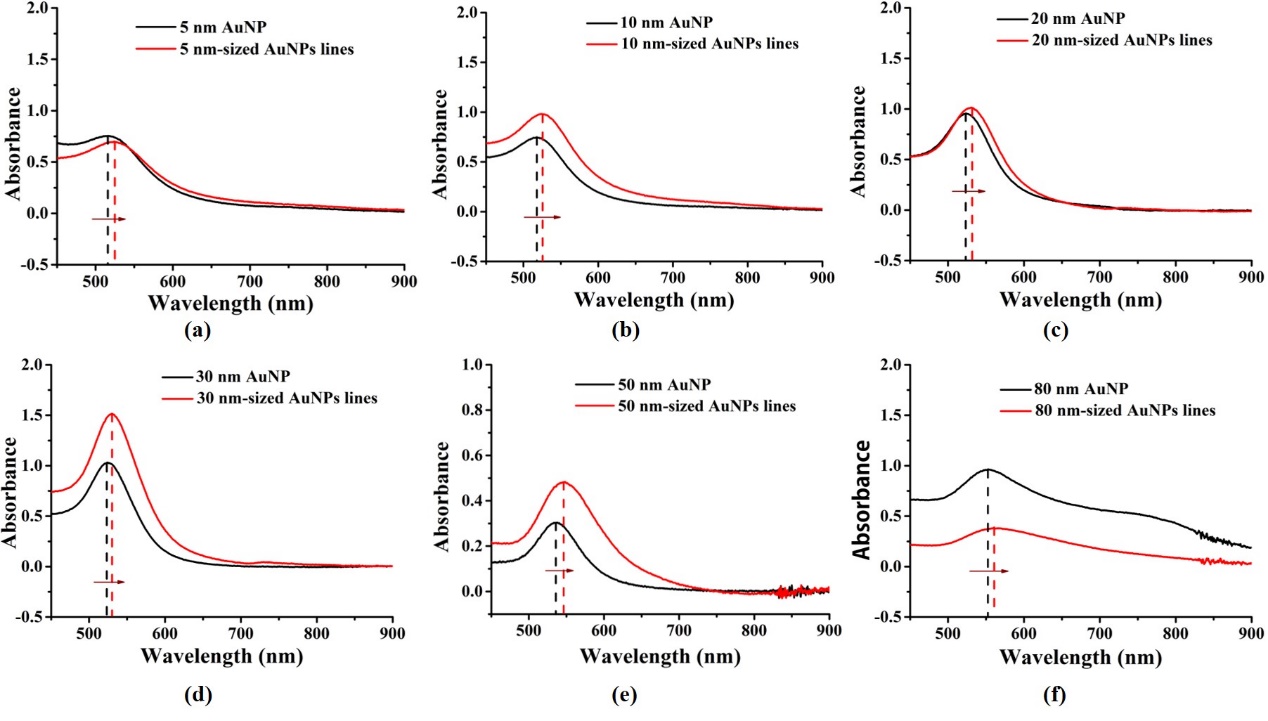


**FIGURE S11.** UV–Vis Characterization of different sized AuNPs lines.

**Sequences of Oligonucleotides Used in This Work.**ssDNA_1_: TTTTTTTTTTTTTTT AGCGA-SH

**For assembly of 1D RDNs (left to right 5’-3’):**

S_1_: CAGGGCTGGGCATAGAAGTCAGGGCAGAGACGAGTTGAGAATACGAGT

S_2_: TGAGAATACGAGTTGAGAATCCGACCATTGTGCGCTATCTTCATCTTA

N_1_: TCTCAACT TCAACTCG TATTCTCA ACTCGTAT

N_2_: CCCTGACT CACAATGG TCGGATTC CGTCTCTG

N_3_: AAAAAAAAAAAAAAA CAGCCCTG TAAGATGA AGATAGCG TCTATGCC

**For assembly of 2D DNA lattices (left to right 5’-3’):**

S_3_: GAGATCCAGCATTCACAGGGCTGGGCATAGAAGT CAGGGCAGAGAC

GAGTTGAGAATACGAGTAGAATGCGAACTGGT

S_4_: TGAATGCTGGATCTCTGAGAATACGAGTTGAGAATCCGACCATTGTGCG

CTATCTTCATCTTAACCAGTTCGCATTCT

N_2_: CCCTGACT CACAATGG TCGGATTC CGTCTCTG

N_3_: AAAAAAAAAAAAAAACAGCCCTGTAAGATGAAGATAGCGTCTATGCC

N_4_: AAAAAAAAAAAAAAATCTCAACTTCAACTCGTATTCTCAACTCGTAT
